# Visualization of Gastric Adenocarcinoma Lymph Node Metastases by Microscopy with Ultraviolet Surface Excitation

**DOI:** 10.17691/stm2024.16.6.03

**Published:** 2024-12-27

**Authors:** G.M. Denisenko, Y.M. Valieva, S.E. Solovyeva, N.B. Serejnikova, V.A. Petrov, G.S. Budylin, P.S. Timashev, A.L. Fayzullin

**Affiliations:** Junior Researcher, Laboratory of Clinical Biophotonics, Institute for Regenerative Medicine; I.M. Sechenov First Moscow State Medical University (Sechenov University), 8/2 Trubetskaya St., Moscow, 119991, Russia; Student, N.V. Sklifosovsky Institute of Clinical Medicine; I.M. Sechenov First Moscow State Medical University (Sechenov University), 8/2 Trubetskaya St., Moscow, 119991, Russia; MD, PhD, Head of the Pathology Department, Scientific and Clinical Center No.1; B.V. Petrovsky Russian Research Center of Surgery, 2 Abrikosovskiy Lane, Moscow, 119991, Russia; PhD, Leading Researcher, Laboratory of Digital Microscopic Analysis, Institute for Regenerative Medicine; I.M. Sechenov First Moscow State Medical University (Sechenov University), 8/2 Trubetskaya St., Moscow, 119991, Russia; PhD, Junior Researcher, Laboratory of Clinical Biophotonics, Institute for Regenerative Medicine; I.M. Sechenov First Moscow State Medical University (Sechenov University), 8/2 Trubetskaya St., Moscow, 119991, Russia; PhD, Head of the Laboratory of Clinical Biophotonics, Institute for Regenerative Medicine; I.M. Sechenov First Moscow State Medical University (Sechenov University), 8/2 Trubetskaya St., Moscow, 119991, Russia; DSc, Associate Professor, Director of the Biomedical Science & Technology Park; I.M. Sechenov First Moscow State Medical University (Sechenov University), 8/2 Trubetskaya St., Moscow, 119991, Russia; MD, PhD, Head of the Laboratory of Digital Microscopic Analysis, Institute for Regenerative Medicine; I.M. Sechenov First Moscow State Medical University (Sechenov University), 8/2 Trubetskaya St., Moscow, 119991, Russia

**Keywords:** MUSE, microscopy with ultraviolet surface excitation, biophotonics, lymph node metastases, oncology

## Abstract

The detection of lymph node metastases is crucial in oncopathology, as it makes it possible to determine the TNM stage, to design a treatment plan, and predict the survival for cancer patients. The current gold standard for this process is hematoxylin and eosin staining. However, new alternative methods leveraging the unique optical properties of tissue structures are being developed for rapid intraoperative or postoperative application.

**The aim of the study** is to evaluate the effectiveness of identifying lymph node metastases using microscopy with ultraviolet surface excitation (MUSE).

**Materials and Methods:**

17 lymph nodes from the Sechenov University archive (Russia) collected intraoperatively from 6 patients with gastric cancer have been investigated.

In this study, we utilized a MUSE optical system consisting of three UV light-emitting diodes (265 nm) and the Axio Scope A1 microscope (Carl Zeiss, Germany) with various objectives. We introduced a novel combination of fluorescent dyes — Nile red and Hoechst — that had not been previously used with MUSE.

**Results:**

The combination of fluorescent dyes yielded high-contrast images with blue-stained nuclei and orange-to-red stained cytoplasm, effectively visualizing gastric adenocarcinoma cells characterized by abundant cytoplasmic components and large polymorphic nuclei. The presence of irregularly shaped cavities, formed by adenocarcinoma metastases, was also detectable by MUSE.

**Conclusion:**

Biophotonics provides alternative methods for tissue imaging. However, traditional methods are still unsurpassed in the accuracy of detecting cancer metastases and other pathologies. Further refinement of imaging protocols and expanded research into other cancer types are needed to make methods like MUSE applicable for intraoperative diagnosis.

## Introduction

Current pathological diagnosis is based on sectioning and staining patient tissues. Most histological staining techniques, including the classical hematoxylin and eosin (H&E) protocol, are conducted on thin cryo- or formalin-fixed paraffin-embedded (FFPE) sections (4–10 μm) of biological tissues. The preparation of histological slides includes several time-consuming steps: fixation in formalin, histological processing, paraffin embedding, and microtomy [1]. Conventional histological techniques provide high-quality study samples but can occupy approximately 48 h and demand lab assistants with special professional skills.

An important process in oncopathology is the detection of lymph node metastases. The TNM stage [2], scheme of treatment, and survival prediction for cancer patients depend on the number and localization of lymph nodes with metastases [3]. The gold standard for diagnosing is H&E staining of lymph node tissue samples obtained by biopsy or dissections [4, 5].

Recently, new alternative methods of imaging lymph node metastases have been transferred from physics to the workplaces of medical practitioners. The industry is driving to develop slide-free methods without long sample preparation for rapid real-time intraoperative or noninvasive diagnosis. By detecting differences in the optical properties of tissue structures, these techniques can differentiate between normal and tumorous tissues. These methods work on different principles. For example, optical coherence tomography is a non-destructive and high-resolution *in vivo* imaging technology that utilizes light interference to reveal the internal microstructural features of tissues [6]. Magnetic resonance microscopy is a noninvasive imaging technique based on the occurrence of a nuclear magnetic resonance signal that makes it possible to form images of objects with a spatial resolution in the micrometer range [7]. Physical principles of multiphoton microscopy are based on the interaction between a biological target — either a fluorophore or object molecules — and at least two photons from a laser source of different wavelengths [8]. Since multiphoton absorption and consequent fluorescence occur only in the beam waist formed by the microscope objective lens, it becomes possible to separate objects with a submicron resolution. This technique is well suited for tissue imaging and *in vivo* or 3D visualization of biological specimens. Raman spectroscopy is a method of molecular spectroscopy based on the interaction of light with matter, which provides information about molecular vibrations that can be used for molecule identification and estimation of their concentrations [9, 10]. These and other alternative methods provide informative images of tissues and give an opportunity to detect lymph node metastases without sample processing; however, all these methods are highly specific and expensive.

In this study, we used the microscopy with ultraviolet (UV) surface excitation (MUSE) as a novel method of visualization. Richard Levenson and his colleagues are considered to be the founders of this method. His team was the first to apply MUSE for histological analysis [11]. They proposed a novel slide-free technique that significantly contributed to the development of fluorescent tissue imaging. The scientists designed a compact smartphone microscope called Pocket MUSE as a promising tool for pathologists [12]. Recently, Levenson’s team developed a proof-of-concept 3D printed millifluidic histopathology lab-on-a-chip device based on MUSE to automatically handle, process, and image fresh core needle biopsies [13].

This method opens new opportunities in histology and pathology. MUSE allows obtaining high-contrast and informative images due to lack of signals from deep layers. It is a simple and cost-effective, fluorescencebased, slide-free optical imaging system [11]. In a number of articles, this method was used to visualize the microstructure of normal organs such as nerves [14], skin [15], cerebellum, spinal cord, liver, kidney, and prostate [11, 16, 17]. Several articles demonstrated the imaging of tumor margins for skin and breast cancer [15, 18], rhabdomyosarcoma [11], pancreatic and lung adenocarcinomas, papillary thyroid and renal cell carcinomas [11]. The choice of dyes in the protocols depended on the type of tissue and the purpose of the microscopy and included rhodamine and Hoechst [11, 14, 17], eosin and propidium iodide [15], eosin and Hoechst [16], simple eosin [18], TbCl3 solution, and 4′,6-diamidino-2-phenylindole (DAPI) [19].

A lymph node is a small organ in the lymphatic system with a specific structure that can be fully visualized by MUSE. As a proof-of-concept study, MUSE was applied to visualize gastric cancer metastases in FFPE sections stained with TbCl3 solution and DAPI [19]. It was proposed that MUSE can be potentially applied for a rapid lymph node visualization taking several minutes after surgical removal. However, a robust protocol and special optic system are required in order to prove the possibility of detecting metastases and to calculate the time it takes to discover them by MUSE in fresh and unprocessed lymph nodes.

**The aim of our study** was to visualize the microstructure of normal and metastatic lymph nodes without tissue processing by MUSE for improvement and acceleration of the metastasis detection.

In our work, we used a 3D-printed adapter with a set of three UV LEDs and proposed a novel combination of contrast dyes for MUSE: Nile red and Hoechst. Our original approach allowed us to identify pathological morphological features for metastases of adenocarcinomas on a cellular level.

## Materials and Methods

### Clinical specimens

Written informed consent was obtained from all patients. 17 lymph nodes, collected during surgery on patients with gastric cancer (6 patients), were acquired from the Sechenov University archive (Russia).

### Tissue preparation

Each lymph node was cut into 2–3-mm-thick slices, which were stored in 10% neutral buffered formalin. One slice of each lymph node underwent standard histological processing and was embedded in a paraffin block. Paraffin sections with a thickness of 4 μm were prepared and stained with H&E. We scanned the samples with Leica Aperio AT2 (Leica Biosystems, Germany) at 20× magnification. All whole slide images (WSI) were anonymized and did not contain labels referring to clinical cases. The evaluation was done using .svs format WSI files in CaseViewer (3DHistech, Hungary).

### MUSE staining protocol

Formalin-fixed samples were washed in PBS with Tween 20 for 5 min. The average size of the samples was 20×20 mm. Each slice was fluorescently stained with a solution of Nile red (72485-1G; Sigma-Aldrich, USA; working concentration 400 μg/ml) and Hoechst 33258 (B1155; Sigma-Aldrich, USA; working concentration 50 μg/ml) for 5 min. All samples were washed in PBS with Tween 20 for 1 min and then placed on histological slides for visualization.

### Optical design and components

**Figure 1.**
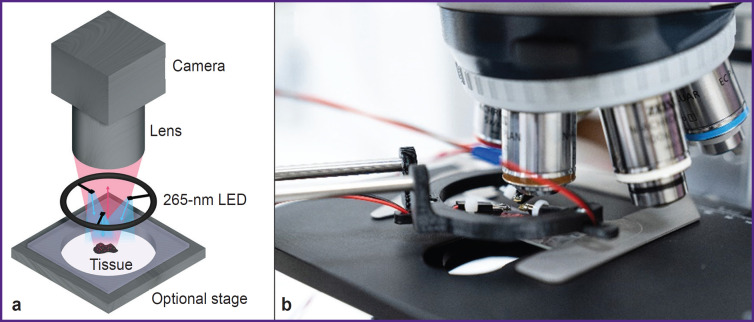
Experimental setup for MUSE (a) and additional 3D adapter for uniform illumination of the sample and registration of fluorescence with a light microscope (b)

The optical system for MUSE imaging comprised three UV light-emitting diodes (LEDs) at 265 nm and the light microscope (Axio Scope A1; Carl Zeiss, Germany) with 2.5x/0.07Na, 5x/0.15Na, and 10x/0.25Na (N-Achroplan; Carl Zeiss, Germany) objectives. The set of UV LEDs was located on a 3D-printed adapter. Such a construction allowed the movement of diodes above the surface of the sample to facilitate the focusing procedure (Figure 1).

Oblique UV excitation light illuminated the specimen, bypassing the glass microscope lens. It served as an intrinsic excitation filter that blocked backscattered UV light from the optical path, as it was opaque in the sub-300 nm spectral region. The oblique excitation angle, compared with full *en face* illumination, could also generate shading across the face of a specimen that usefully highlighted tissue surface topography. To make illumination more uniform over the entire sample surface and to optimize shading in case of complex surface topography, three LEDs were used to illuminate the sample. Fluorescence emission was detected with a digital camera (AXIOCAM 506; Carl Zeiss, Germany), which obtained full-color images of the sample tissue surface. The image acquisition parameters were manually set with Carl Zeiss ZEN software. All samples were studied by two experienced pathologists. We used the program AutoStitch for Windows to make whole section images.

## Results

### Morphological study

**Figure 2.**
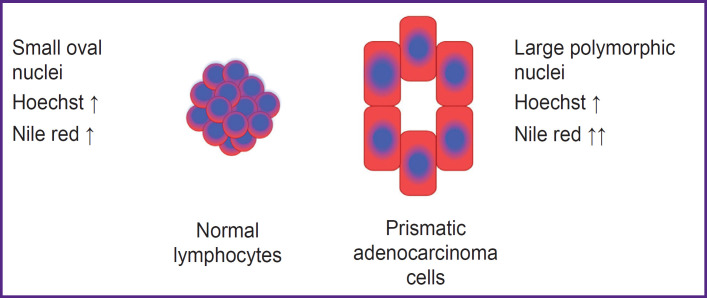
Nile red and Hoechst staining. Normal lymphocytes and prismatic adenocarcinoma cells Lymphocytes with oval blue Hoechst-positive nuclei and faint narrow Nile redpositive cytoplasm are packed together (arrow indicates high expression of fluorescent dye). Tumor cells contained larger polymorphic Hoechst-positive nuclei and significantly more evident Nile red-positive cytoplasm (higher level of expression is indicated by double arrows)

When stained with Hoechst and Nile red and visualized by MUSE, the nuclei stained with Hoechst appeared blue while the cytoplasm gave red or orange fluorescence. It is known that Nile red stains intracellular lipids in cytoplasm [20]. As a result, normal lymphocytes fluorescence almost blue due to few cytoplasmic components, whereas adenocarcinoma cells have abundant cytoplasmic components in addition to the nuclei [19]. Such cancer cells gave radiant fluorescence as large cells with blue nuclei and visible red cytoplasm (Figure 2).

### Normal lymph node

**Figure 3.**
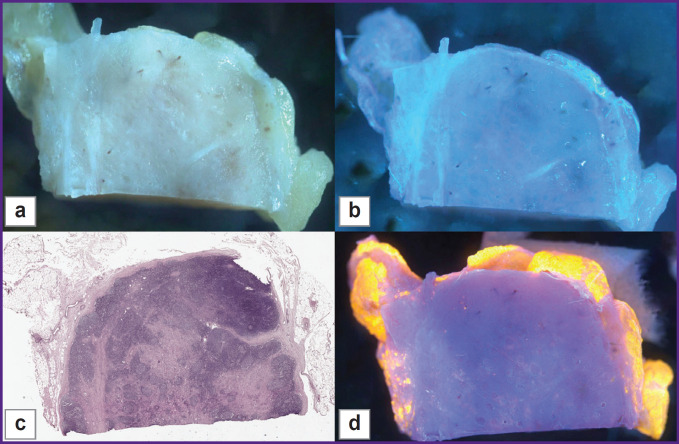
Room light (a), UV-light image without fluorescence dyes (b), H&E (c), MUSE (d) images of normal lymph node Adipose tissue, follicles, and blood vessels are not well visible on the samples without fluorescent dyes (a), (b). A clear difference is seen between lymphoid tissue (dark blue) and surrounding adipose tissue (orange) on the MUSE image (d). Similar tissue structures with fine details were visualized by MUSE (d) and on the H&E-stained slide (c)

**Figure 4.**
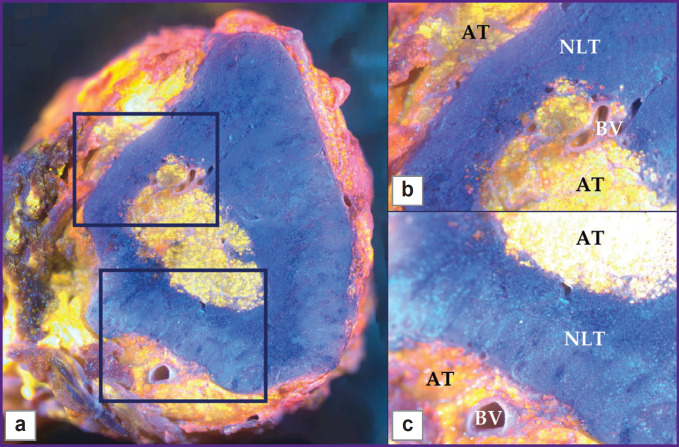
Normal lymph node, MUSE image Whole section (a), 25× magnification (b), (c). Adipose tissue (AT), normal lymph tissue (NLT), blood vessels (BV)

We visualized the surface of normal lymph node slices in 3 different modes and compared the images to WSI. Unstained samples looked smooth and shining under the microscope and were uninformative in room light (Figure 3 (a)). Tissue structures were not clearly visible. The 3D-printed adapter with a set of three UV LEDs provided ultraviolet light, but unstained tissue looked similar to the tissue in room light (Figure 3 (b)). After the application of fluorescent dyes, adipose tissue, lymphoid follicles, blood vessels, and other tissue structures were visible in detail (Figure 4) and matched the H&E image (Figure 3 (c), (d)).

**Figure 5.**
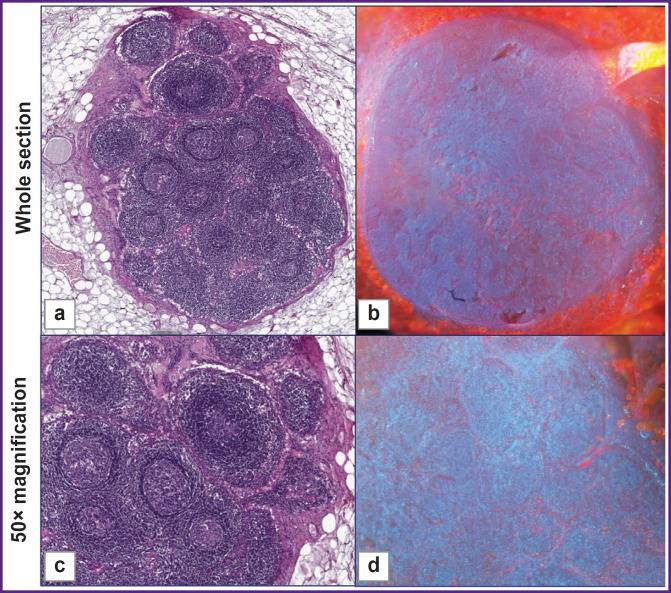
Normal lymph node H&E: encapsulated lymphatic organ surrounded by capsule and adipose tissue. The parenchyma of the node is composed of a mass of lymphatic follicles (a). The lymphocytes are densely packed close to each other in bundles of cells, which form larger follicles (c). MUSE: even surface with oval lymphoid follicles. The adipose tissue surrounding the lymph node was stained orange (b). Lymphoid follicles are visualized as blue structures. The bulk of tissue is visualized as groups of blue ovals indicating densely packed lymphocytes (d)

Normal lymph nodes were examined by MUSE (Figure 5). The lymph nodes had an oval shape. MUSE images were compared with H&E images. H&E images showed typical histological structure of normal lymphatic tissue with specific details, such as adipose tissue, capsule, lymphatic follicles, stroma, and blood vessels (Figure 5 (a), (c)). The surrounding adipose tissue of the lymph node appeared orange on MUSE images after staining, while the majority of the tissue consisted of densely packed blue ovals, indicating a high concentration of densely packed lymphocytes. The lymph node surface was predominantly blue, since lymphocytes have relatively large nuclei and a thin rim of the cytoplasm. The pattern of packing and specific spatial orientation of lymphocytes was universal for the whole section of the lymph node. Thin interlayers of Nile red-positive stromal components that corresponded to the medulla of the lymph node were observed. Blood vessels were visualized as cavities with a thin lining giving red fluorescence. We observed cells and endothelium of the inner surface of the blood vessels by focusing on regions below the section. However, red fluorescence was relatively rare in normal lymph nodes and did not spread diffusively among parenchymal parts of the node (Figure 5 (b), (d)).

### Metastatic lymph node

**Figure 6.**
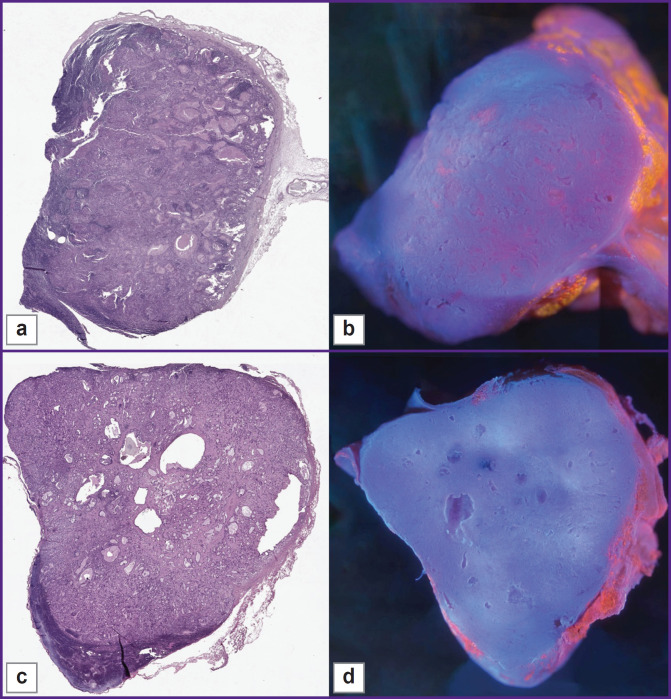
Lymph nodes with gastric adenocarcinoma metastases, whole sections H&E: adenocarcinoma metastases are glandular structures with a homogeneous eosinophilic substance or with a cavity inside (a), (c). A clear border between normal tissue (dark tissue) and tumor (light tissue with glandular structures) (c). MUSE: the surface area of normal follicular structure is significantly reduced due to the invasion of metastases or such areas are completely absent. There are a large number of cavities on the surface of the lymph node (b), (d)

We examined lymph nodes with gastric adenocarcinoma metastases (Figure 6). The lymph node with metastases had an uneven, irregular shape due to the glandular nature of the tumor.

**Figure 7.**
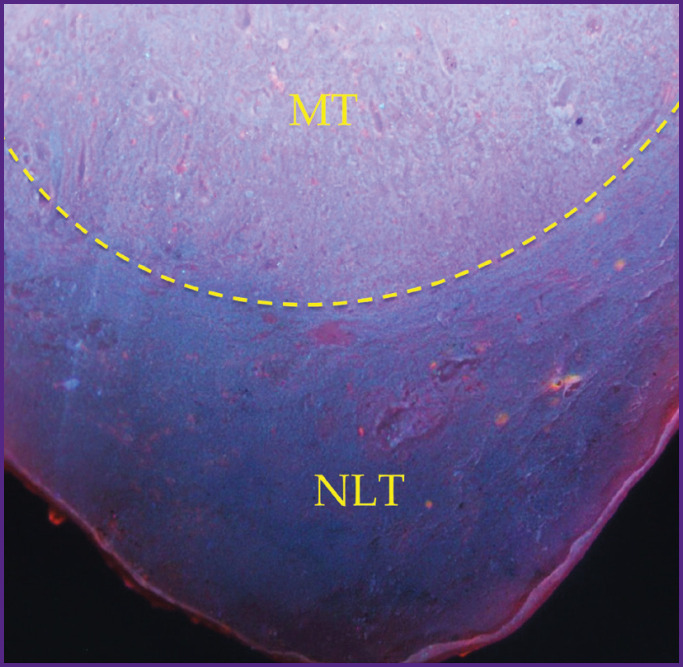
A clear difference between normal lymph tissue and metastases, 25× magnification The tissue with metastases (MT) is above the normal lymph tissue (NLT)

The main structures, such as areas of adipose tissue, uniform areas of typical lymphoid tissue, and large metastases, were similar on H&E and MUSE. On H&E images, adenocarcinoma metastases contained glandular structures with prismatic epithelium. These glandular structures were visualized as cavities, which included homogeneous eosinophilic substances, mucous or secretion, or were empty. Normal lymphatic tissue and areas of metastases were separated by clear borders (Figure 7). In some cases, metastases replaced the entire mass of normal tissue, and this border was absent (see Figure 6). On the MUSE whole image, the presence of cavities was an outstanding feature of the lymph node with metastases. Large and irregularly shaped cavities were formed by adenocarcinoma metastases. Such cavities corresponded to the glandular structure or foci of necrosis in the relevant H&E images. They could include mucus or cell debris. This feature enabled visualization of adenocarcinoma metastases at low magnification (Figure 6 (b), (d)). Other cavities visualized by MUSE were blood or lymphatic vessel lumens.

**Figure 8.**
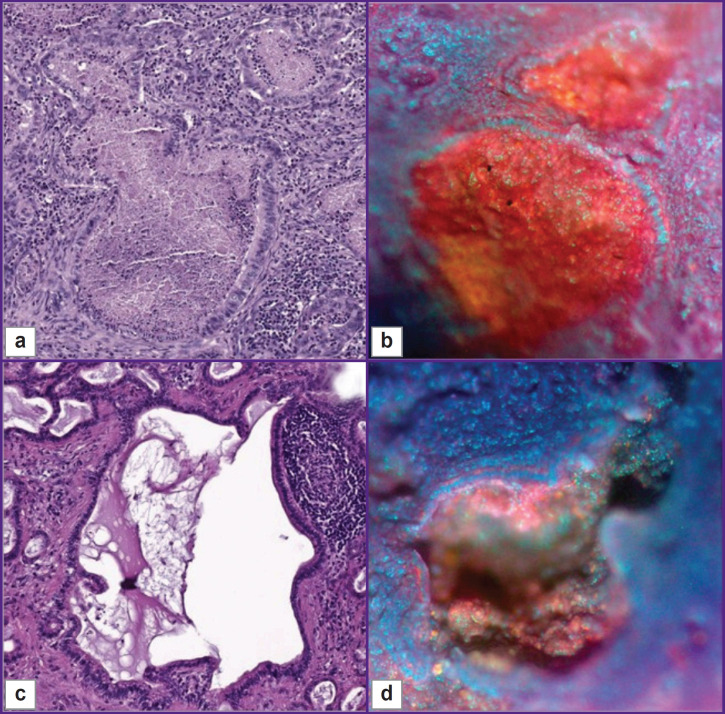
Lymph nodes with gastric adenocarcinoma metastases, 100× magnification H&E: metastases are atypical structures consisting of large prismatic cells. These glandular structures are surrounded by a mass of normal lymphocytes. Inside the metastases there is a homogeneous eosinophilic substance (a), (c). MUSE: cavities lined by large prismatic cells. The inner surface of the cavities had a red tint due to the predominance of epithelial cells. They had small pleomorphic blue nuclei and a well-defined red cytoplasm (b), (d)

Cavities were lined with large prismatic cells on MUSE at 100× magnification (Figure 8). These tumor cells were larger in size than typical lymphocytes. The inner surface of the cavities had a red tint due to the predominance of epithelial cells. They had small pleomorphic blue nuclei and a well-defined red cytoplasm. The areas with normal lymphocytes surrounded the cavities, but their typical packing was disrupted. Also, small areas of the normal lymphatic tissue remained in the sample. The normal tissue contained small cells with blue fluorescence, whereas the metastatic cavities had surfaces with red fluorescence. There was a clear border between the normal tissue and the tumor in some cases (see Figure 7), where the area with metastases was small. Most often, this border was absent due to the metastasis spreading through the whole area of the lymph node.

## Discussion

Tissue examination by MUSE has the potential to become part of routine diagnosis for pathologists. This method has a number of advantages compared to other existing methods of tissue visualization. Firstly, MUSE allows one to explore tissues very rapidly. The entire process, including formalin fixation, staining with fluorescence dyes, washing with PBS, and microscopy, takes about 10–15 min. Secondly, this method is easy to use and doesn’t demand special professional skills. The ability to stain tissue samples without lengthy preliminary preparation is a significant advantage of MUSE.

MUSE allowed us to evaluate the lymph nodes on three levels of observation. At low magnification, abnormalities on the surface of adenocarcinoma became evident. Cavities of mucin and necrotic detritus were identified and corresponded to the presence of cancerous cells. MUSE images revealed clearly a significant difference in Nile red fluorescence between the normal lymphoid and metastatic tissues. Finally, MUSE allowed us to focus on groups of cells and assess their packing, fluorescence, and size. Combined together, these findings can form the basis for future guidelines for using MUSE in practice.

Besides the advantages, MUSE has several limitations. This method provides tissue imaging, but the resolution of MUSE is lower than that of traditional histological methods. Further research is required to improve the image quality of MUSE. This could be achieved by using new combinations of dyes that are more specific to intracellular structures, such as membranes or deposits. Additionally, one sample cannot be stained repeatedly. Fluorescent dyes irreversibly bind with cellular structures and lose fluorescence over time due to exposure to light. Subsequent staining does not provide the previously observed fluorescence. However, it is important to note that even repeated staining with fluorescent dyes does not affect further standard staining methods such as H&E.

We have chosen lymphatic nodes as the subject of our study because there are small organs most commonly affected by metastases. MUSE has already been used to detect lymph node metastases [19]. In this study, the FFPE sections of lymph nodes from gastric cancer patients were stained with fluorescent dyes (DAPI and a solution containing TbCl3). Representative images with histological structures that were similar to those seen in H&E images have been obtained. The pathologists detected metastatic cancer cells using MUSE. Additionally, fluorescent images exhibited more distinct boundaries between cancer cells and normal lymph node tissues compared to H&E images.

We used MUSE to visualize the lymph node samples fixed with 10% formalin without preliminary sample preparation. The avoidance of tissue processing allowed using MUSE to investigate the samples in 3D and real-time.

The visualization of metastases in lymph nodes was not intuitive and required a lot of comparisons with relevant H&E images. We used MUSE to examine lymph nodes with gastric adenocarcinoma metastases. This type of cancer is common, and its metastases typically have a glandular structure. MUSE visualized only large metastases and was significantly less sensitive to solid and small areas of tumor cells.

The accuracy of MUSE analysis depends on a set of factors. The type of metastases is an important factor for MUSE. It is possible to image large heterogeneous structures by MUSE, such as adenocarcinoma metastases. An even section of a tissue sample is another significant factor. If the tissue surface is rough, a normal lymph node can present signs of pathology. Laboratory assistants should make even sections for MUSE examination.

We propose several tips for staining protocols for researchers interested in MUSE. The combination of dyes and their specificity affect MUSE results. Dyes should bind to different tissue structures and should contrast well. There are two staining principles: using fluorescent dyes sequentially or combining them into a single solution. In the first case, one fluorescence signal dominates the stained sample. In the second case, the tissue is stained more evenly; smaller metastases are more visible, and prismatic cells with blue nuclei and red cytoplasm are more representative at 100× magnification. The dye solution should be freshly prepared to avoid staining with a weak fluorescence signal. The concentrations of dyes in the solution can depend on the type of tissue and should not overstain it leaving excess dye in the samples. Moreover, the solution should always contain the same concentration for certain tissue types to ensure consistent interpretation of results.

## Conclusion

Our results underscore the potential of using MUSE as an alternative method of cancer visualization. The imaging of lymph nodes by MUSE can allow the detection of metastases without time-consuming tissue processing. The accuracy of this method is lower compared to traditional methods. Not all tissue structures are visualized by MUSE, but this method can be used for real-time diagnosis, for example, as an alternative to cryosectioning. The most prominent advantage of MUSE is instant visualization, which completely eliminates the time gap between a surgical manipulation and pathologist verification. With the help of MUSE, it is possible to determine the presence or absence of metastases in lymph nodes. However, if the area of metastases is small or has a homogeneous structure, the pathologist may miss a metastasis and make an incorrect diagnosis. In this case, this method cannot replace traditional histological methods completely and requires additional investigations to improve MUSE imaging, such as the implementation of computer vision. In order to make MUSE applicable to intraoperative diagnosis, we need to improve our protocols for imaging and expand the research to other types of cancer in the following studies.

## Data Availability

The relevant data generated and/ or analyzed in the current study is available from the corresponding author upon reasonable request.
